# Exome-wide assessment of the functional impact and pathogenicity of multinucleotide mutations

**DOI:** 10.1101/gr.239756.118

**Published:** 2019-07

**Authors:** Joanna Kaplanis, Nadia Akawi, Giuseppe Gallone, Jeremy F. McRae, Elena Prigmore, Caroline F. Wright, David R. Fitzpatrick, Helen V. Firth, Jeffrey C. Barrett, Matthew E. Hurles, on behalf of the Deciphering Developmental Disorders study

**Affiliations:** 1 Wellcome Sanger Institute, Wellcome Genome Campus, Hinxton, CB10 1SA, United Kingdom;; 2 Division of Cardiovascular Medicine, Radcliffe Department of Medicine, University of Oxford, Oxford, OX3 9DU, United Kingdom;; 3 Institute of Biomedical and Clinical Science, University of Exeter Medical School, Exeter, EX2 5DW, United Kingdom;; 4 MRC Human Genetics Unit, MRC IGMM, University of Edinburgh, Western General Hospital, Edinburgh EH4 2XU, United Kingdom;; 5 Department of Clinical Genetics, Cambridge University Hospitals NHS Foundation Trust, Cambridge, CB2 0QQ, United Kingdom

## Abstract

Approximately 2% of de novo single-nucleotide variants (SNVs) appear as part of clustered mutations that create multinucleotide variants (MNVs). MNVs are an important source of genomic variability as they are more likely to alter an encoded protein than a SNV, which has important implications in disease as well as evolution. Previous studies of MNVs have focused on their mutational origins and have not systematically evaluated their functional impact and contribution to disease. We identified 69,940 MNVs and 91 de novo MNVs in 6688 exome-sequenced parent–offspring trios from the Deciphering Developmental Disorders Study comprising families with severe developmental disorders. We replicated the previously described MNV mutational signatures associated with DNA polymerase zeta, an error-prone translesion polymerase, and the APOBEC family of DNA deaminases. We estimate the simultaneous MNV germline mutation rate to be 1.78 × 10^−10^ mutations per base pair per generation. We found that most MNVs within a single codon create a missense change that could not have been created by a SNV. MNV-induced missense changes were, on average, more physicochemically divergent, were more depleted in highly constrained genes (pLI ≥ 0.9), and were under stronger purifying selection compared with SNV-induced missense changes. We found that de novo MNVs were significantly enriched in genes previously associated with developmental disorders in affected children. This shows that MNVs can be more damaging than SNVs even when both induce missense changes, and are an important variant type to consider in relation to human disease.

In genomic analyses, single-nucleotide variants (SNVs) are often considered independent mutational events. However, SNVs are more clustered in the genome than expected if they were independent (Seidman et al. 1987; Amos 2010; Michaelson et al. 2012). On a finer scale, there is an excess of pairs of mutations within 100 bp that appear to be in perfect linkage disequilibrium in population samples (Stone et al. 2012; Harris and Nielsen 2014; Ségurel et al. 2014). Although some of this can be explained by the presence of mutational hotspots, natural selection, or compensatory mechanisms, it has been shown that multinucleotide mutations play an important role (Schrider et al. 2011). Recent studies found that 2.4% of de novo SNVs were within 5 kb of another de novo SNV within the same individual (Besenbacher et al. 2016) and that 1.9% of de novo SNVs appear within 20 bp of another de novo SNV (Schrider et al. 2011). Multinucleotide variants (MNVs) occurring at neighboring nucleotides are the most frequent of all MNVs (Besenbacher et al. 2016). Moreover, analysis of phased human haplotypes from population sequencing data also showed that nearby SNVs are more likely to appear on the same haplotype than on different haplotypes (Schrider et al. 2011).

The mutational origins of MNVs are not as well understood as for SNVs; however, different mutational processes leave behind different patterns of DNA change that are dubbed mutational “signatures.” Distinct mutational mechanisms have been implicated in creating MNVs. Polymerase zeta is an error-prone translesion polymerase that has been shown to be the predominant source of de novo MNVs in adjacent nucleotides in yeast (Harris and Nielsen 2014; Zhu et al. 2015; Besenbacher et al. 2016). The most common mutational signatures associated with polymerase zeta in yeast have also been observed to be the most common signatures among MNVs in human populations (Harris and Nielsen 2014) and were also found to be the most prevalent in de novo MNVs in parent–offspring trios (Besenbacher et al. 2016). It has been suggested that translesion DNA polymerases play an important role in the creation of MNVs more generally (Chen et al. 2015; Zhu et al. 2015). A distinct mutational signature has also been described that has been attributed to the action of APOBEC deaminases (Alexandrov et al. 2013).

Although MNVs are an important source of genomic variability, their functional impact and the selection pressures that operate on this class of variation has been largely unexplored. In part, this is because of many commonly used workflows for variant calling and annotation of likely functional consequence annotating MNVs as separate SNVs (Sandmann et al. 2017). When the two variants comprising an MNV occur within the same codon—as occurs frequently given the propensity for MNVs at neighboring nucleotides—interpreting MNVs as separate SNVs can lead to an erroneous prediction of the impact on the encoded protein. The Exome Aggregation Consortium (ExAC) systematically identified and annotated more than 5000 MNVs that occurred within the same codon in genes, including some within known disease-associated genes (Lek et al. 2016). Although individual pathogenic MNVs have been described (ClinVar; http://www.ncbi.nlm.nih.gov/clinvar/), the pathogenic impact of MNVs as a class of variation is not yet well understood.

Here, we analyzed 6688 exome-sequenced parent–offspring trios from the Deciphering Developmental Disorders (DDD) Study to evaluate systematically the strength of purifying selection acting on MNVs in the population sample of unaffected parents, and to quantify the contribution of pathogenic de novo MNVs to developmental disorders in the children.

## Results

### Identifying and categorizing MNVs

**Figure 1.**
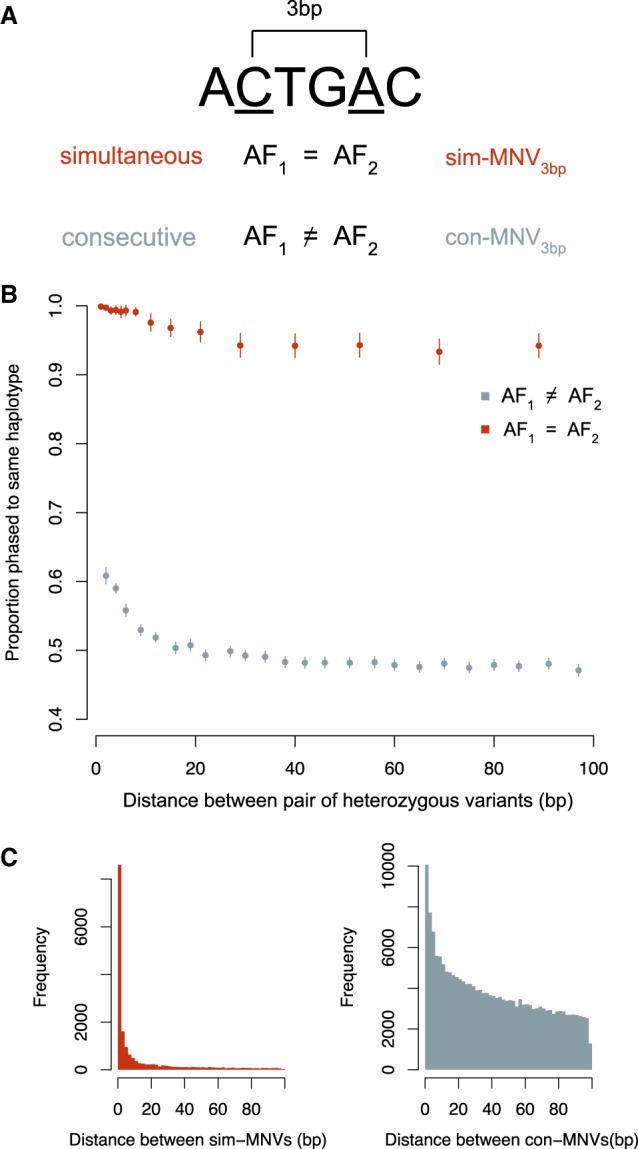
Properties of MNVs. (*A*) Schematic showing how sim-MNVs, two variants that occur simultaneously, are defined as having two variants with identical allele frequencies, and con-MNVs, two variants that occur consecutively, as having different allele frequencies. (*B*) Proportion of pairs of heterozygous variants (possible MNVs) that phase to the same haplotype as a function of distance separated by sim and con. (*C*) The number of sim-MNVs and con-MNVs by distance between the two variants.

We identified 69,940 MNVs transmitted from the 13,376 unaffected trio parents as well as 91 de novo MNVs in the trio children. We defined MNVs as comprising two variants within 20 bp of each other that phased to the same haplotype across >99% of all individuals in the data set in which they appear (Fig. 1A). This definition encompasses both MNVs because of a single mutational event and MNVs in which one SNV occurs after the other. The variants were phased using trio-based phasing, which meant that the ability to phase the variants was not dependent on the distance between them, and it also provided an additional layer of quality assurance by conditioning on the variant being called in both parent and child. MNVs tend to have lower mapping quality than SNVs, and so, traditional variant filtering criteria based on quality metrics would potentially miss a substantial number of MNVs. This also enabled us to use the same filtering criteria for different classes of variants to ensure comparability. The distance of 20 bp between variants was selected as we observed that pairs of SNVs that define potential MNVs are only enriched for phasing to the same haplotype at this distance (Fig. 1B). De novo MNVs were defined as two de novo SNVs within 20 bp of each other and were confirmed to be on the same haplotype using read-based phasing. Because of the small numbers, we were able to filter these by manually inspecting these variants using the Integrative Genomics Viewer (IGV) (Robinson et al. 2017). Ten of the de novo MNVs fell within genes previously associated with dominant developmental disorders. These were all validated experimentally using MiSeq or capillary sequencing.

Different mutational mechanisms are likely to create MNVs at different distances. To capture these differences, we stratified analyses of mutational spectra based on distance between the variants. The distance between the two variants that make up an MNV will be denoted as a subscript. For example, adjacent MNVs will be referred to as MNV_1bp_. MNVs can be created either by a single mutational event or by consecutive mutational events. For MNVs that were created by a single mutational event, the pair of variants are likely to have identical allele frequencies as they are unlikely to occur in the population separately (we assume recurrent mutations and reversions are rare). The proportion of nearby pairs of SNVs with identical allele frequencies that phase to the same haplotype remains close to 100% even at a distance of 100 bp apart (Fig. 1B). We can assume that these variants most likely arose simultaneously and will be referred to as sim-MNVs. The proportion of pairs of SNVs with different allele frequencies that phase to the same haplotype approaches 50% at around 20 bp. These probably arose consecutively and will be referred to as con-MNVs. We observed that sim-MNVs account for 19% of all MNVs and 53% of MNV_1bp_. All de novo MNVs are, by definition, sim-MNVs as they occurred in the same generation.

We identified 888 trinucleotide variants (trinucleotide sim-MNVs) that we defined as three SNVs within 20 bp with identical allele frequencies. One hundred fourteen of these occurred in three adjacent nucleotides. We observed one de novo trinucleotide MNV.

### Analysis of MNV mutational spectra confirms mutational origins

Differences in mutational spectra across different subsets of MNVs can reveal patterns or signatures left by the underlying mutational mechanism. We analyzed the spectra of both simultaneous and consecutive MNV_1bp_, MNV_2bp_, and MNV_3–20bp_. For sim-MNVs, the proportion of variants that fell into these groups were 51%, 12%, and 37%, respectively. For con-MNVs, most variants were further away with the proportions being 10%, 7%, and 83% (Table 1; Fig. 1C). We observed significant differences between the mutational spectra of sim-MNVs and con-MNVs (Supplemental Fig. S1A,C).

**Table 1.**
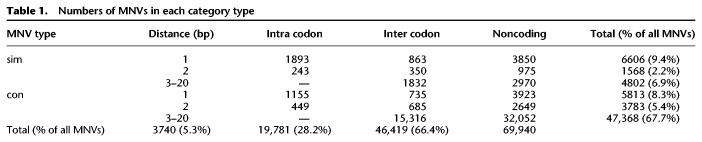
Numbers of MNVs in each category type

DNA polymerase zeta, a translesion polymerase, is a known frequent source of de novo MNVs and has been associated with the mutational signatures GC → AA and GA → TT (Harris and Nielsen 2014; Besenbacher et al. 2016). These signatures, and their reverse complements, account for 22% of all sim-MNV_1bp_s (Supplemental Fig. S1B). These two signatures made up 18% of the de novo sim-MNV_1bp_s, which is comparable to the 20% of observed de novo MNVs in a recent study (Supplemental Fig. S2B; Besenbacher et al. 2016). In the remaining 78% of sim-MNV_1bp_s, we observed 16 other mutations, after Bonferroni multiple correction, that were significantly more prevalent in sim-MNV_1bp_s compared with con-MNV_1bp_s. This suggests that there are other unidentified mechanisms that are specific to creating sim-MNVs. The most prevalent sim-MNV_1bp_ that is not attributed to polymerase zeta is TC > AT, which accounts for 4% of all sim-MNV_1bp_s. We observed two de novo sim-MNV_1bp_s with this signature; however, an extensive literature search has not yielded any possible mechanism behind this mutation.

APOBEC are a family of cytosine deaminases that are known to cause clustered mutations in exposed stretches of single-stranded DNA. These mutational signatures are commonly found in cancer and more recently discovered in germline mutations (Roberts et al. 2013; Pinto et al. 2016). The most common mutation for sim-MNV_2bp_ is CnC → TnT, where n is the intermediate base between the two mutated bases, and is ∼8% of mutations (Supplemental Fig. S1C). They are found primarily in a TCTC> TTTT or CCTC> CTTT sequence context (Supplemental Fig. S1D). CC and TC are known mutational signatures of APOBEC (Alexandrov et al. 2013; Harris 2013; Pinto et al. 2016). However, the APOBEC signature described previously in germline mutations was found in pairs of variants that were a larger distance apart (10–50 bp). C…C → T…T was also the most prolific mutation in sim-MNV_3–20bp_ and had a significantly larger proportion of APOBEC motifs in both variants compared with con-MNV_3–20bp_ (*P*-value 0.0056) (Supplemental Fig. S1E). The mutation C…C → T…T was the most frequent de novo MNV_2–20bp_ (Supplemental Fig. S2C). However, only three of the 12 de novo MNV_2–20bp_ had APOBEC motifs.

There were six other mutations that are significantly more common in sim-MNV_2bp_ compared with con-MNV_2bp_. The most prevalent of these is CnG > TnT, which accounts for 3% of sim-MNV_2bp_. We did not observe any de novo MNVs with this mutation, and we were not able to attribute a mutational mechanism after reviewing the literature.

We analyzed the mutational signatures of the set of 114 adjacent trinucleotide sim-MNVs and found that the most prevalent mutation was AAA > TTT (Supplemental Fig. S3); however, we were not able to establish a possible mutational mechanism.

Mutational signatures in con-MNVs were primarily driven by CpG sites. In humans, the 5′ C in a CpG context is usually methylated and has a mutation rate that is approximately 10-fold higher than any other context (Duncan and Miller 1980). For con-MNV_2–30bp_, the most common mutation is C…C → T…T and is driven by two mutated CpG sites CG…CG > TG…TG (S1d). For con-MNV_1bp_s, 24% are accounted for by the mutation CA → TG, and its reverse complement (S1b). These adjacent consecutive mutations most likely came about owing to a creation of a CpG site by the first mutation. If the first mutation creates a CpG, then the mutations would be expected to arise in a specific order: CA > CG> TG. We would therefore expect that the A > G mutation would happen first and that variant would have a higher allele frequency than the subsequent C > T. This was the case for 96% of the 1445 CA > TG con-MNV_1bp_s. This was also the case for 96% and 92% of the other less common possible CpG creating con-MNVs CC > TG and AG > CA. CA > TG is probably the most common variant as it relies on a transition mutation A > G happening first, which has a higher mutation rate compared with the transversions C > G and T > G. We identified 255 de novo con-MNVs, and 26 of these were de novo con-MNV_1bp_s. In half of these, the inherited variant created a CpG site, which was then mutated de novo in our data.

We also observed that for con-MNV_1–3bp_s that were not as a result of CpG creating sites, the first variant increases the mutability of the second variant more than expected by chance. We compared the median difference in mutation probability of the second variant based on the heptanucleotide sequence context before and after the first variant occurred using a Wilcoxon signed-rank test (Aggarwala and Voight 2016). The median increase in mutation probability of the second variant was 0.0002 (Wilcoxon signed-rank test *P*-value 9.8 × 10^−17^).

### Misannotation of MNVs

When an MNV occurs within a single codon, the consequence of this MNV can be different to the consequences when the two comprising variants are annotated separately. We found that for ∼98% of the intra-codon MNVs that we identified, the consequence class (synonymous, missense, stop-gained, etc.) of the MNV was the same as at least one of the SNVs annotated separately. For only 1% of the intra-codon MNVs was the consequence class of the MNV more severe than the separate SNVs. For almost all of these, the MNV caused a stop-gain. Most intra-codon MNVs result in a missense change (Table 2), and so, even though one of the comprising variants is most likely annotated as a missense separately as well, the MNV can create a different amino acid change.

**Table 2.**
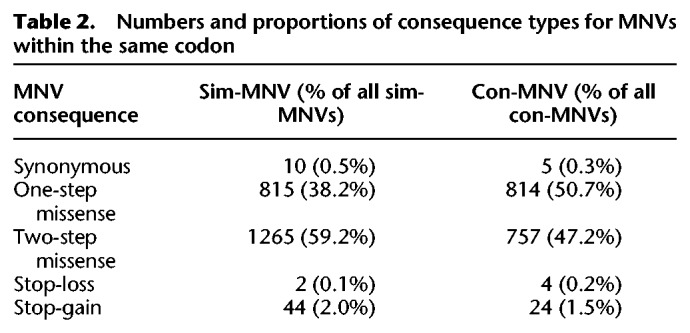
Numbers and proportions of consequence types for MNVs within the same codon

### Functional consequences of MNVs

**Figure 2.**
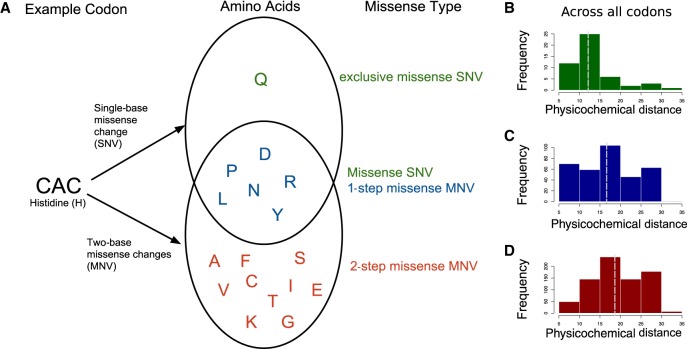
Classification of intra-codon MNV missense mutations. (*A*) Example of how one-step missense MNVs and two-step missense MNVs are classified using a single codon “CAC.” Venn diagram shows amino acids that can be created with either a single-base change or a two-base change in the codon “CAC.” (*B*–*D*) Across all codons, the distribution of physicochemical distances for the amino acid changes caused by different types of missense variants: (*B*) exclusive SNV missense; (*C*) one-step MNV missense; and (*D*) two-step MNV missense. Dashed line indicates the median of the distribution.

The structure of the genetic code is not random. The code has evolved such that the codons that correspond to amino acids with similar physicochemical properties are more likely to be separated by a single-base change (Wong 1975; Amirnovin 1997). SNVs that result in a missense change will only alter one of the bases in a codon; however, MNVs that alter a single codon (“intra-codon” MNVs) will alter two of the 3 bp. Therefore, they are more likely to introduce an amino acid that is further away in the codon table and thus less similar physicochemically to the original amino acid. Most intra-codon MNVs result in a missense change (Table 2). Intra-codon missense MNVs can be classified into two groups: “one-step” and “two-step” missense MNVs. One-step missense MNVs lead to an amino acid change that could also have been achieved by an SNV, whereas two-step MNVs generate amino acid changes that could only be achieved by two SNVs. For example, if we consider the codon CAC that codes for histidine (H), then a single-base change in the codon can lead to missense changes, creating seven possible amino acids (Y, R, N, D, P, L, Q) (Fig. 2A). There are one-step missense MNVs within that codon that can lead to most of the same amino acids (Y,R,N,D,P,L). However, two-step missense MNVs could also lead to an additional 11 amino acids that could not be achieved by an SNV (F, S, C, I, T, K, S, V, A, E, G). For some codons, there are also amino acid changes that can only be created by a single-base change, for this histidine codon this would be glutamine (Q). These will be referred to as exclusive SNV missense changes. For this analysis, we only considered sim-MNVs that most likely originated from the same mutational event. This is because we were primarily interested in the functional effects of mutations occurring simultaneously and in which the amino acid produced would have changed directly from the original amino acid to the MNV consequence and not via an intermediate amino acid.

### MNVs can create a missense change with a larger physicochemical distance compared with missense SNVs

We assessed the differences in the amino acid changes between exclusive missense SNVs, one-step MNVs, and two-step MNVs by examining the distribution of physicochemical distance for each missense variant type across all codons (Fig. 2B). We used a distance measure between quantitative descriptors of amino acids based on multidimensional scaling of 237 physical–chemical properties (Venkatarajan and Braun 2001). We chose this measure as it does not depend on observed substitution frequencies, which may create a bias owing to the low MNV mutation rate making these amino acid changes inherently less likely. We found that the median amino acid distance was significantly larger for two-step missense MNVs compared with one-step missense MNVs (Wilcoxon signed-rank test, *P*-value 1.10 × 10^−7^). The median distance for one-step missense MNVs was also significantly larger from exclusive SNV missense changes (Wilcoxon signed-rank test, *P*-value 0.0008) (Fig. 2B–D).

### Missense MNVs are on average more damaging than missense SNVs

**Figure 3.**
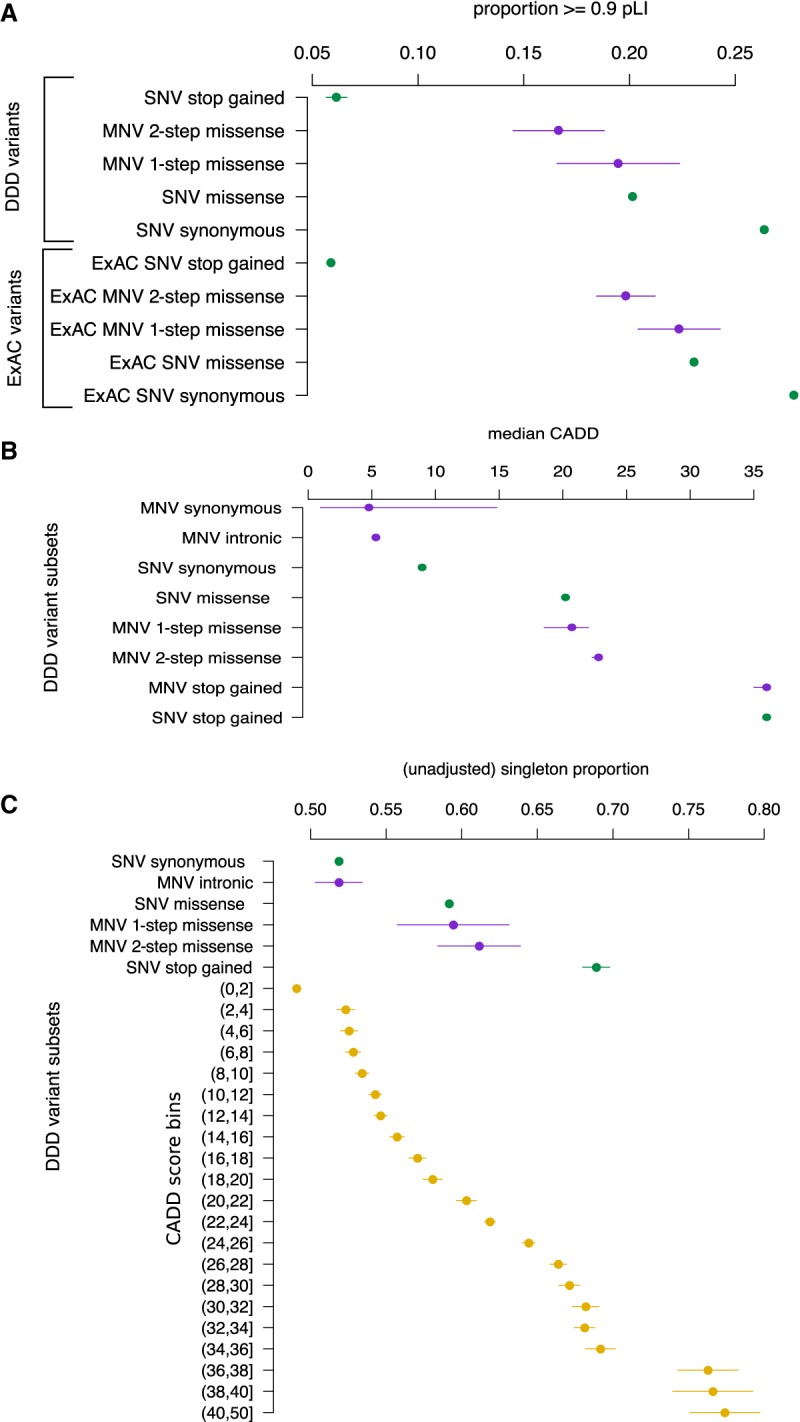
Quantifying the pathogenicity of MNVs. (*A*) Proportion of variants that fall in genes with pLI ≥ 0.9 over different classes of variants for both DDD and ExAC data sets. SNVs are green; MNVs, purple. Lines are 95% confidence intervals. (*B*) The median CADD score over different classes of variants identified from DDD data with bootstrapped 95% confidence intervals. (*C*) Singleton proportion for different classes of DDD variants. In yellow are SNVs stratified by binned CADD scores with their corresponding singleton proportions. Lines are 95% confidence intervals.

If the physicochemical differences between these classes of missense variants resulted in more damaging mutations in the context of the protein, then we would expect to see a greater depletion of two-step missense MNVs compared with one-step missense MNVs or missense SNVs in highly constrained genes. We looked at the proportion of variants of different classes that fell in highly constrained genes, as defined by their intolerance of truncating variants in population variation, as measured by the probability of loss-of-function intolerance (pLI) score (Fig. 3A). Highly constrained genes were defined as those with a pLI score ≥0.9 (Samocha et al. 2014). MNVs that impact two nearby codons (inter-codon MNVs) are likely to have a more severe consequence on protein function, on average, than an SNV impacting on a single codon. We observed that the proportion of inter-codon MNV_1–20bp_s that fall in highly constrained genes (pLI > 0.9) is significantly smaller compared with missense SNVs (*P*-value 0.0007) (Fig. 3A). For intra-codon MNVs, we saw that the proportion of two-step missense MNVs observed in highly constrained genes was also significantly smaller than for missense SNVs (*P*-value 0.0016). The proportion of one-step missense MNVs was not significantly different from either missense SNVs or two-step missense MNVs. The analysis was repeated using SNVs and MNVs that were identified by the ExAC, which were subject to different filtering steps (Lek et al. 2016). The same relationship was observed as the proportion of ExAC two-step MNVs in high pLI genes was significantly smaller than for ExAC missense SNVs (*P*-value 9.84 × 10^−6^).

We then compared variant deleteriousness across the variant classes using a combined annotation-dependent depletion (CADD) score that integrates many annotations such as likely protein consequence, constraint, and mappability (Fig. 3B; Kircher et al. 2014). We found that the median CADD score for two-step missense MNVs was significantly higher than both one-step missense MNVs (Wilcoxon signed-rank test, *P*-value 0.00017) and missense SNVs (Wilcoxon signed-rank test, *P*-value 2.70 × 10^−8^). Two-step MNV missense had a median CADD score of 22.8 compared with a one-step missense median CADD score of 20.7 and a SNV missense median CADD score of 20.2.

The proportion of singletons across variant classes is a good proxy for the strength of purifying selection acting in a population (Lek et al. 2016). The more deleterious a variant class, the larger the proportion of singletons. We found that the singleton proportion for two-step missense MNVs was nominally significantly higher compared with missense SNVs (*P*-value 0.02) (Fig. 3C). The increase in proportion corresponded to an increase of about two in the interpolated CADD score. This is concordant with the increase in CADD scores that was computed directly above.

### Estimation of the MNV mutation rate

We estimate the genome-wide mutation rate of sim-MNV_1–20bp_ to be 1.78 × 10^−10^ mutations per base pair per generation by scaling the SNV mutation rate based on the relative ratio of segregating polymorphisms for MNVs and SNVs (Watterson 1975). For this estimate, we only used variants that fell into nonconstrained genes (pLI < 0.1) and noncoding regions to avoid any bias from selection. We assume that recurrent mutation is insufficiently frequent for both classes of variation to alter the proportionality between the number of segregating polymorphisms and the mutation rate. This estimate is ∼1.6% of the mutation rate estimate for SNVs and accords with the genome-wide proportions of SNVs and MNVs described previously (Schrider et al. 2011). We were concerned that the selective pressure on MNVs and SNVs would still be different in nonconstrained genes and that this might affect our mutation rate estimate. To see if this was the case, we applied the same method to estimate the SNV missense mutation rate across coding region, and found that our estimate was concordant with that obtained from using an SNV trinucleotide context mutational model (Samocha et al. 2014).

We also estimated the MNV mutation rate using the set of de novo MNVs that fell into nonconstrained genes (pLI < 0.1) that have not previously been associated with dominant developmental disorders, and obtained a concordant mutation rate estimate of 1.79 × 10^−10^ (confidence interval 0.88, 2.70 × 10^−10^) mutations per base pair per generation, very similar to the estimate based on segregating polymorphisms described above.

### Contribution of de novo MNVs to developmental disorders

**Figure 4.**
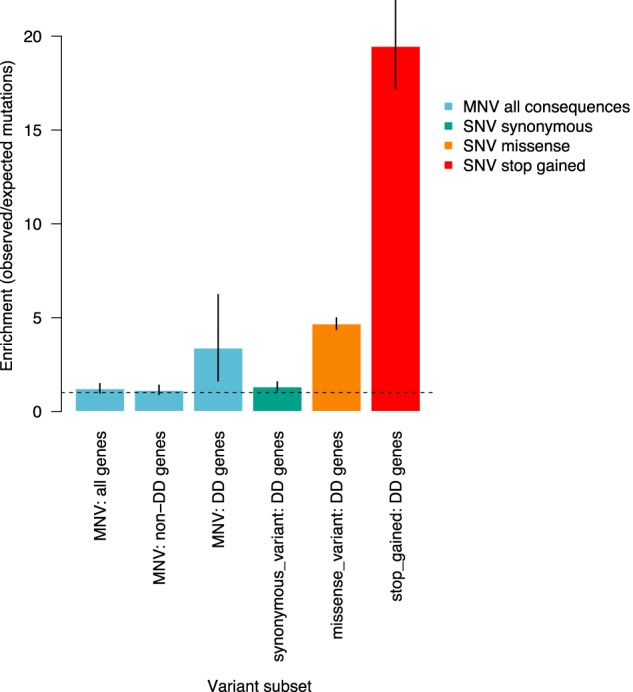
Enrichment of de novo MNVs in DDD study. Ratio of observed number of de novo MNVs versus the expected number of de novo MNVs based on the estimate of the MNV mutation rate. Compared with enrichment of SNVs in DD genes in consequence classes synonymous, missense, and stop-gain.

We identified 10 de novo MNVs within genes known to be associated with dominant developmental disorders (DD-associated) in the DDD trios (Table 3), which is a significant (Poisson test, *P*-value 1.03 × 10^−3^) 3.7-fold enrichment compared with what we would expect based on our estimated MNV mutation rate. This enrichment is similar in magnitude to that observed for de novo SNVs in the same set of DD-associated genes (Fig. 4). We evaluated whether DD-associated genes are enriched for the primary mutagenic dinucleotide contexts associated with the signatures of polymerase zeta to ensure this observation was not driven by sequence context. We found that DD-associated genes had a small (1.02-fold) but significant (proportion test, *P*-value 1.9 × 10^−59^) enrichment of polymerase zeta dinucleotide contexts compared with genes not associated with DD. However, this subtle enrichment is insufficient to explain the fourfold enrichment of de novo MNVs in these genes. The enrichment for de novo MNVs remains significant after correcting for sequence context (Poisson test, *P*-value 2.28 × 10^−3^).

**Table 3.**
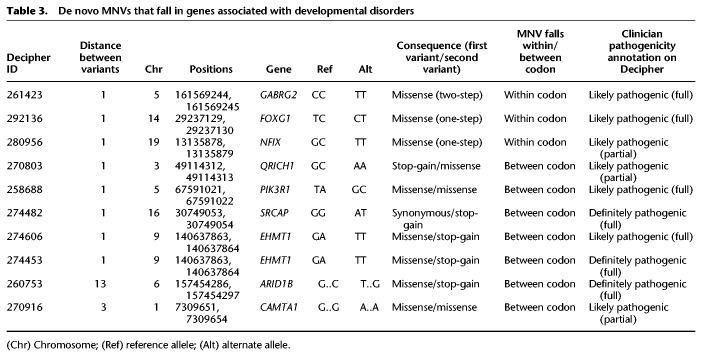
De novo MNVs that fall in genes associated with developmental disorders

(Chr) Chromosome; (Ref) reference allele; (Alt) alternate allele.

Eight of the 10 de novo MNVs in DD-associated genes were 1 bp apart, whereas the other two were 3 and 13 bp apart. All of these de novo MNVs were experimentally validated in the child (and their absence confirmed in both parents) using either MiSeq or capillary sequencing. All 10 MNVs are thought to be pathogenic by the child's referring clinical geneticist. Seven of the MNVs impacted two different codons, whereas three fell within the same codon, one of which created a two-step missense change. Of those MNVs that impacted two codons, five caused a premature stop codon. We found a recurrent de novo MNV in the gene *EHMT1* in two unrelated patients that bore the distinctive polymerase zeta signature of GA > TT.

### De novo MNVs are underrepresented in clinically reported variants in DD-associated genes

To assess whether de novo MNVs are being underreported in genes associated with DD, we downloaded all clinically reported variants in DD-associated genes from ClinVar (accessed September 2017) (Landrum et al. 2014; http://www.ncbi.nlm.nih.gov/clinvar/). We looked at the number of intra-codon missense MNVs in genes that have at least one reported pathogenic missense mutation. This was to ensure that missense mutations in that gene would likely cause DD. We focused on intra-codon MNVs as it is the interpretation of this class of MNV that is most impacted by failing to consider the variant as single unit. We calculated the expected number of pathogenic de novo MNVs in these genes based on the MNV mutation rate and the number of pathogenic SNV missense variants reported. We observed a significant depletion of only 24 reported pathogenic de novo MNVs compared with the expected number of 52 across 321 genes (Poisson test, *P*-value 2.8 × 10^−5^).

We also looked for clinically relevant SNVs in ClinVar that overlapped with population sim-MNVs that we identified in our data. We found one SNV that had been reported as a stop-gain variant in the gene *AGPAT2*. The variant had been reported as pathogenic and of uncertain significance for congenital generalized lipodystrophy type 1 by two contributors. However, we observe this variant as an MNV in our data set in three individuals. The MNV falls within the same codon and causes a missense as opposed to a stop-gain, which decreases its likelihood of pathogenicity.

### MNV mutator phenotype

Five individuals had more than one de novo sim-MNV. This is significantly greater than what we would expect assuming these arose independently. Using our estimated MNV mutation rate, the probability of seeing five or more individuals in our data set with more than one MNV is 5.8 × 10^−7^. The number of MNVs per person range from two to five de novo MNVs. These mostly appear on different chromosomes and have different distances between the pair of variants. None of the MNVs share the same mutation, and the number of mutations is too small to pick up on more subtle similarities in the mutational signatures. A comparable mutator phenotype has been observed in other classes of genetic variation such as CNVs, but, similarly, a relevant mutational mechanism has not yet been discovered (Liu et al. 2017). A larger number of de novo MNVs will help to uncover possible mechanisms behind the mutator phenotype.

## Discussion

MNVs constitute a unique class of variant, in terms of both mutational mechanism and functional impact. We found that 18% of segregating MNVs were at adjacent nucleotides. We estimated that 19% of all MNVs represent a single mutational event, increasing to 53% of MNV_1bp_. We estimated the sim-MNV germline mutation rate to be 1.78 × 10^−10^ mutations per base pair per generation, ∼1.6% that of SNVs. Most population genetics models assume that mutations arise from independent events (Harris and Nielsen 2014). MNVs violate that assumption, and this may affect the accuracy of these models. Recent studies suggest that certain phylogenetic tests of adaptive evolution incorrectly identify positive selection when the presence of these clustered mutations is ignored (Venkat et al. 2018). Correcting these population genetic models will require knowledge of the rate and spectrum of MNV mutations. The observation of a possible MNV mutator phenotype complicates this correction further. We replicated the observations from previous studies that several different mutational processes underlie MNV formation and that these tend to create MNVs of different types. Error-prone polymerase zeta predominantly creates sim-MNV_1bp_ (Harris and Nielsen 2014; Besenbacher et al. 2016). APOBEC-related mutation processes have been described to generate MNVs in the range of 10–50 bp (Alexandrov et al. 2013; Harris 2013; Roberts et al. 2013), but here we show that an enrichment for APOBEC motifs can be detected down to MNV_2bp_. Nonetheless, there remain other sim-MNVs that cannot be readily explained by either of these mechanisms, and it is likely that other, less distinctive, mutational mechanisms remain to be delineated as catalogs of MNVs increase in scale. These future studies should also investigate whether these MNV mutational signatures differ subtly between human populations as has been recently observed for SNVs (Harris 2015). Consecutive MNVs, in contrast, show greater similarity with known SNV mutation processes, most notably with the creation and subsequent mutation of mutagenic CpG dinucleotides. The non-Markovian nature of this consecutive mutation process challenges Markovian assumptions that are prevalent within standard population genetic models (Rizzato et al. 2016).

Our findings validated the intuitive hypothesis that MNVs that impact upon two codons within a protein are likely, on average, to have a greater functional impact than SNVs that alter a single codon. We evaluated the functional impact of intra-codon MNVs using three complementary approaches: (1) depletion within genes under strong selective constraint, (2) shift toward rarer alleles in the site frequency spectrum, and (3) enrichment of de novo mutations (DNMs) in known DD-associated genes in children with DDs. We showed that intra-codon MNVs also tend to have a larger functional impact than SNVs and that MNV missense changes that cannot be achieved by a single SNV are, on average, more deleterious than those that can. This is most likely because they are on average more physicochemically different compared with amino acids created by SNVs and are not as well tolerated in the context of the encoded protein. These “two-step” missense MNVs make up more than half of all sim-MNVs that alter a single codon. We also identified 10 pathogenic de novo MNVs within the DDD study, including both intra-codon and inter-codon MNVs. With larger trio data sets, we will have more power to tease apart more subtle differences in pathogenic burden and purifying selection between different classes of MNVs and SNVs, for example, to test whether two-step missense de novo MNVs are more enriched than missense SNVs or one-step missense MNVs in developmental disorders. More data will also allow us to assess the population genetic properties of inter-codon MNVs.

Our findings emphasize the critical importance of accurately calling and annotating MNVs within clinical genomic testing both to improve diagnostic sensitivity and to avoid misinterpretation. We observed that pathogenic de novo MNVs are significantly underrepresented and possibly misannotated among reported pathogenic clinical variants in ClinVar, indicating that current analytical workflows have diminished sensitivity for identifying pathogenic MNVs. In a recent comparison of eight different variant calling tools, it was noted that only two callers, FreeBayes and VarDict, report two mutations in close proximity as MNVs. The others reported them as two separate SNVs (Sandmann et al. 2017). Both FreeBayes and VarDict are haplotype-aware callers, which is necessary for MNV detection (Garrison and Marth 2012; Lai et al. 2016). Even if variant callers do not identify MNVs directly, software also exists that can correct a list of previously called SNVs to identify misannotated MNVs (Wei et al. 2015). To further our understanding of the role of MNVs in evolution and disease, calling and annotating these variants correctly is a vital step.

## Methods

### Variant and de novo calling in DDD

The analysis in this report was conducted using exome sequencing data from the DDD study of families with a child with a severe, undiagnosed developmental disorder. The recruitment of these families has been described previously (Wright et al. 2015): 7833 trios from 7448 families and 1791 singleton patients (without parental samples) were recruited at 24 clinical genetics centers within the UK National Health Service and the Republic of Ireland. Families gave informed consent to participate, and the study was approved by the UK Research Ethics Committee (10/H0305/83, granted by the Cambridge South Research Ethics Committee, and GEN/284/12, granted by the Republic of Ireland Research Ethics Committee). In this analysis, we only included trios from children with unaffected parents in our analysis to avoid bias from pathogenic inherited MNVs. This was defined as those trios in which the clinicians did not report any phenotypes for either parent. This resulted in a total of 6688 complete trios. Sequence alignment and variant calling of SNV and insertions/deletions were conducted as previously described. De novo mutations were called using DeNovoGear and filtered as previously (Ramu et al. 2013; Deciphering Developmental Disorders 2017).

### Identifying MNVs

MNVs were defined as two nearby variants that always appear on the same haplotype. To identify all possible candidate MNVs, we searched for two heterozygous variants that were within 100 bp of each other in the same individual across 6688 DDD proband VCFs and that had a read depth of at least 20 for each variant. These pairs of variants were classified as MNVs if both variants appeared on the same haplotype for >99% of individuals in which they appear. This was determined by phasing variants using parental exome data. We were able to determine phase for approximately two-thirds of all possible MNVs across all individuals. Those that could not be phased were discarded. Read-based phasing for these variants proved to be more error-prone than trio-based phasing and so was not performed. After examining the properties of these MNVs, we restricted the analyses to those that were 1–20 bp of each other. We identified 69,940 unique MNVs.

A set of 693,837 coding SNVs was obtained from the DDD probands with the exact same ascertainment as those for MNVs (read depth >20, phased to confirm inheritance). These were used when comparing MNV properties to SNVs to reduce any ascertainment bias.

To identify de novo MNVs we looked within a set of 51,942 putative DNMs for pairs of de novo variants within 20 bp of each other. This set of DNMs had been filtered requiring a low minor allele frequency (MAF), low strand bias, and low number parental alt reads. We did not impose stricter filters at this stage as true de novo MNVs tend to have worse quality metrics than true de novo SNVs. We found 301 pairs, ∼1.2% of all candidate DNMs. A third of these were 1–2 bp apart (Fig. 3A). For analysis of mutational spectra, we did not filter these further; however, when looking at functional consequences of these de novo MNVs, we wanted to be more stringent and examined IGV plots for all de novo MNVs, of which 91 passed IGV examination.

### Estimating the MNV mutation rate

We estimated the MNV mutation rate by scaling the SNV mutation rate estimate of 1.1 × 10^−8^ mutations per base pair per generation by the ratio of MNV segregating sites/SNV segregating sites observed in our data set (Roach et al. 2010). This approach is based on a rearrangement of the equation for the Watterson estimator (Watterson 1975). This is outlined below, where *θ* is the Watterson estimator, *μ* is the mutation rate, *K* denotes the number of segregating sites, *N*_*e*_ is the effective population size, *n* is the sample size, and *a*_*n*_ is *n* − 1th harmonic number.

$$\hat{\theta }=\frac{K_{SNV}}{a_{n}}=4N_{e}\;\mu_{SNV},$$

$$\mu_{SNV}=\frac{K_{SNV}}{a_{n}4N_{e}}=1.1\times 10^{-8},$$

$$a_{n}4N_{e}=\frac{K_{SNV}}{1.1\times 10^{-8}},$$

$$\mu_{MNV}=\frac{K_{MNV}}{a_{n}4N_{e}},$$

$$\;=\frac{K_{MNV}}{K_{SNV}}1.1\times 10^{-8}.$$

To avoid any potential bias from selection, we excluded variants that fell into potentially constrained genes (pLI > 0.1). The MNV mutation rate was estimated to be 1.78 × 10^−10^ mutations per base pair per generation.

We estimated the SNV missense mutation rate in the same way by scaling the overall SNV mutation rate by the ratio of the number of missense SNVs in unconstrained genes compared with all SNVs and obtained an estimate of the missense mutation rate across coding regions to be 1.07 × 10^−8^ per coding base pair per generation, which agrees with the estimate of 1.09 × 10^−8^ per coding base per generation, which was calculated using the trinucleotide context mutational model described by Samocha et al. (2014).

### Enrichment of de novo MNVs

To test for the enrichment of de novo MNVs, we used a Poisson test for three categories of genes: all genes, genes known to be associated with developmental disorders, and genes that are not known to be associated with developmental disorders. Genes known to be associated with developmental disorders, in which de novo mutations can be pathogenic, were defined as those curated on the Gene2Phenotype website (http://www.ebi.ac.uk/gene2phenotype/) and were listed as monoallelic that were “confirmed” and “probable” associated with DD. We did the same tests for synonymous, missense, and protein-truncating variants using gene-specific mutations rates for each consequence type derived by Samocha et al. (2014; Supplemental Fig. S4). The significance of these statistical tests was evaluated using a Bonferroni-corrected *P*-value threshold of 0.05/12 to take into account the 12 tests across all three subsets of genes, SNV consequence types, and MNVs (Supplemental Fig. S4). To correct for sequence context when comparing DD genes and non-DD genes, we adjusted the expected number of MNVs in the DD genes category based on the excess of polymerase zeta dinucleotide contexts. We also estimated the MNV mutation rate using all variants, as well as a more stringent estimate just using variants that fell into noncoding regions. When we redid the enrichment analysis using these mutation rate estimates of varying stringency, the enrichment of de novo MNVs in DD-associated genes remained significant (all variants: *P*-value 2.7 × 10^−4^; noncoding control regions: *P*-value 4.9 × 10^−3^) (Supplemental Fig. S5A). The SNV mutation rate estimate varies across studies; therefore, we also recalculated the MNV mutation rates using SNV mutation rate estimates of 1.0 × 10^−8^ and 1.2 × 10^−8^ mutations per base pair per generation (Ségurel et al. 2014). These were also recalculated across the three different variant subsets (all variants, excluding variants in genes with pLI > 0.1, variants in noncoding control regions). The enrichment ratio of de novo MNVs that fall into DD genes ranged from 2.7 to 4.8; however, it always remained significantly greater than one, and the confidence intervals consistently overlapped with that of the SNV missense enrichment ratio (Supplemental Fig. S5B).

### Analysis of the number of clinically reported de novo MNVs

We downloaded all clinically reported variants from the website ClinVar and subsetted these variants to those that fell into autosomal dominant DDG2P genes and those that were annotated as “definitely pathogenic” or “likely pathogenic.” This set was then subsetted to 321 genes with at least one pathogenic missense mutation. This was to ensure that missense mutations cause disease in these genes. We then counted the numbers of SNV missense variants and used this to estimate the number of expected missense MNVs across those genes. This was scaled using the ratio of the SNV to MNV missense mutation rate across these genes. The MNV missense mutation rate was calculated as

$$\begin{array}{r} \mu_{DDG2P\;MNV\;missense}=\;\mu_{MNV}\times \frac{2}{3}\times 0.97\times \sum \\ coding\;bp\;in\;DDG2P\;genes,\; \end{array}$$

where 2/3 is the probability of an MNV falling within a codon and 0.97 is the probability that a within-codon MNV results in a missense change. The expected number of missense MNVs in DDG2P genes was then calculated as follows:

$$Expected\;\#\;reported\;pathogenic\;missense\;MNVs=\#\;reported\;missense\;SNVs\times \frac{\mu_{DDG2P\;MNV\;missense\;}}{\mu_{DDG2P\;SNV\;missense}}.$$

This assumes that the enrichment of MNV and SNV missense mutations in these genes is comparable as we have observed in DDD. This yielded an expected number of 51.94 reported pathogenic MNVs compared with 24 observed reported pathogenic MNVs. To test if this difference was significant, we performed a Poisson test (*P*-value 2.8 × 10^−5^).

All results from statistical tests are summarized in Supplemental Table S1.

## Data access

The raw exome sequencing data from this study have been submitted to the European Genome-phenome Archive (EGA; https://www.ebi.ac.uk/ega/) under accession number EGAS00001000775 and are available following Data Access Committee (DAC) approval.

## Supplementary Material

Supplemental Material
