# Supplementary material for: Exome-wide assessment of the functional impact and pathogenicity of multinucleotide mutations
Source: Genome Res. 2019 Jul;29(7):1047–56. doi: 10.1101/gr.239756.118 (PMC6633265; doi:10.1101/gr.239756.118)
Supplement: Supplemental Material [file supp_29_7_1047__index.html]

Exome-wide assessment of the functional impact and pathogenicity of multinucleotide mutations — Supplemental Material 

# Exome-wide assessment of the functional impact and pathogenicity of multinucleotide mutations

## Supplemental Material

- Supplemental\_material.pdf
