# Supplementary material for: Exome-wide assessment of the functional impact and pathogenicity of multinucleotide mutations
Source: Genome Res. 2019 Jul;29(7):1047–56. doi: 10.1101/gr.239756.118 (PMC6633265; doi:10.1101/gr.239756.118)
Supplement: Supplemental Material [file supp_gr.239756.118_Supplemental_material.pdf]

# Supplemental Material

Figure S1: Mutational spectra of MNVs
Figure S2: Mutational spectra of *de novo* MNVs
Figure S3: Mutational spectra of adjacent trinucleotide sim-MNVs
Figure S4: Extended version of Figure 5
Figure S5: Sensitivity of MNV enrichment analysis to MNV mutation rate estimates

Table S1: Summary of statistical tests performed in the analyses

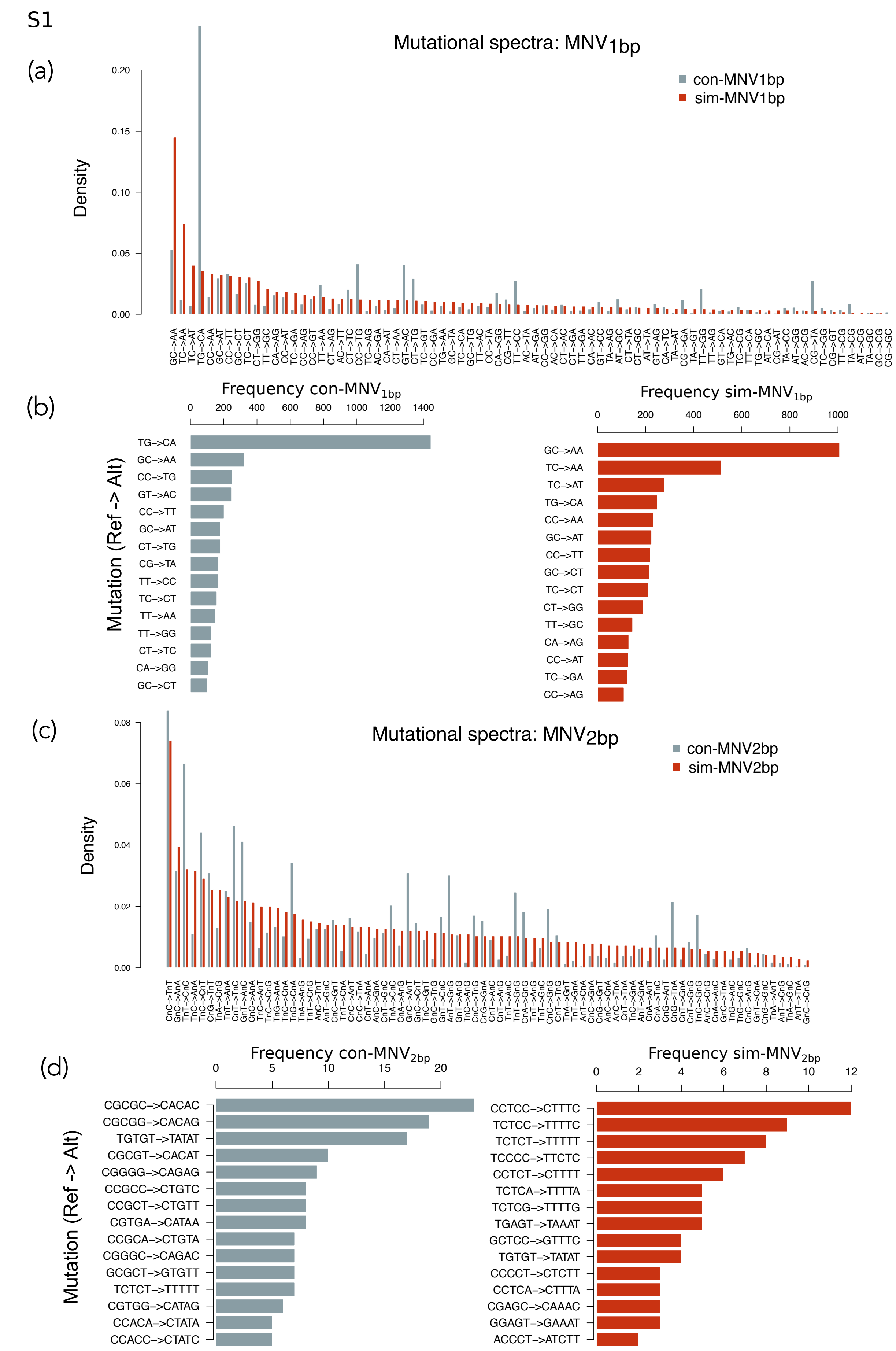


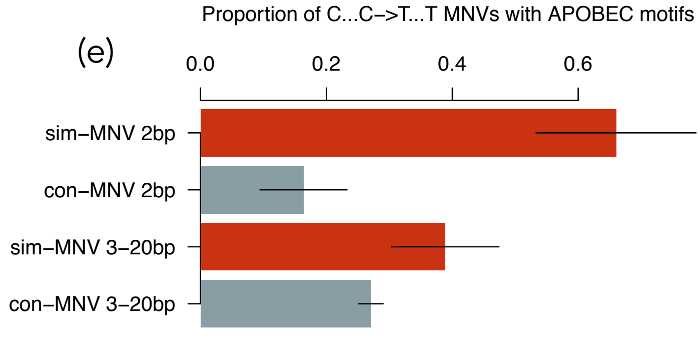


S1: Mutational Spectra of MNVs (a) The frequency of mutational spectra for sim-MNV1bp and con-MNV1bp (b) The 15 most common mutations for sim-MNV1bp and con-MNV1bp (c) The frequency of mutational spectra for sim-MNV2bp and con-MNV2bp (b) The 15 most common mutations for sim-MNV2bp and con-MNV2bp (e) The proportion of C…C->T…T MNVs that have motifs associated with mutations caused by APOBEC.

Figure S2

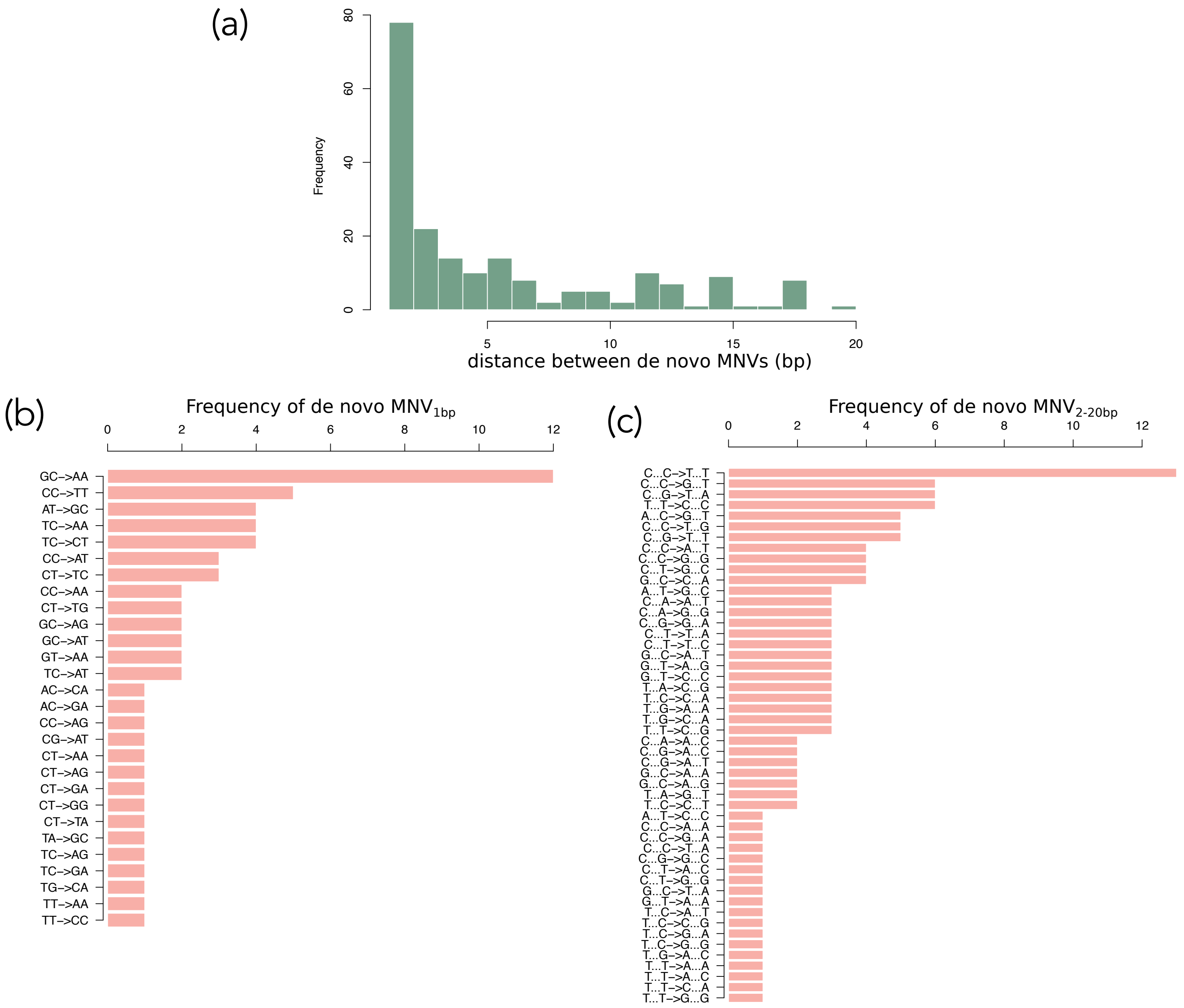


Figure S2: Mutational spectra of de novo MNVs (a) Frequency of de novo MNVs according to the distance between the two variants in base pairs (b) Frequency of different mutation types for de novo MNV1bp (c) Frequency of different mutation types for de novo MNV2-20bp

Figure S3

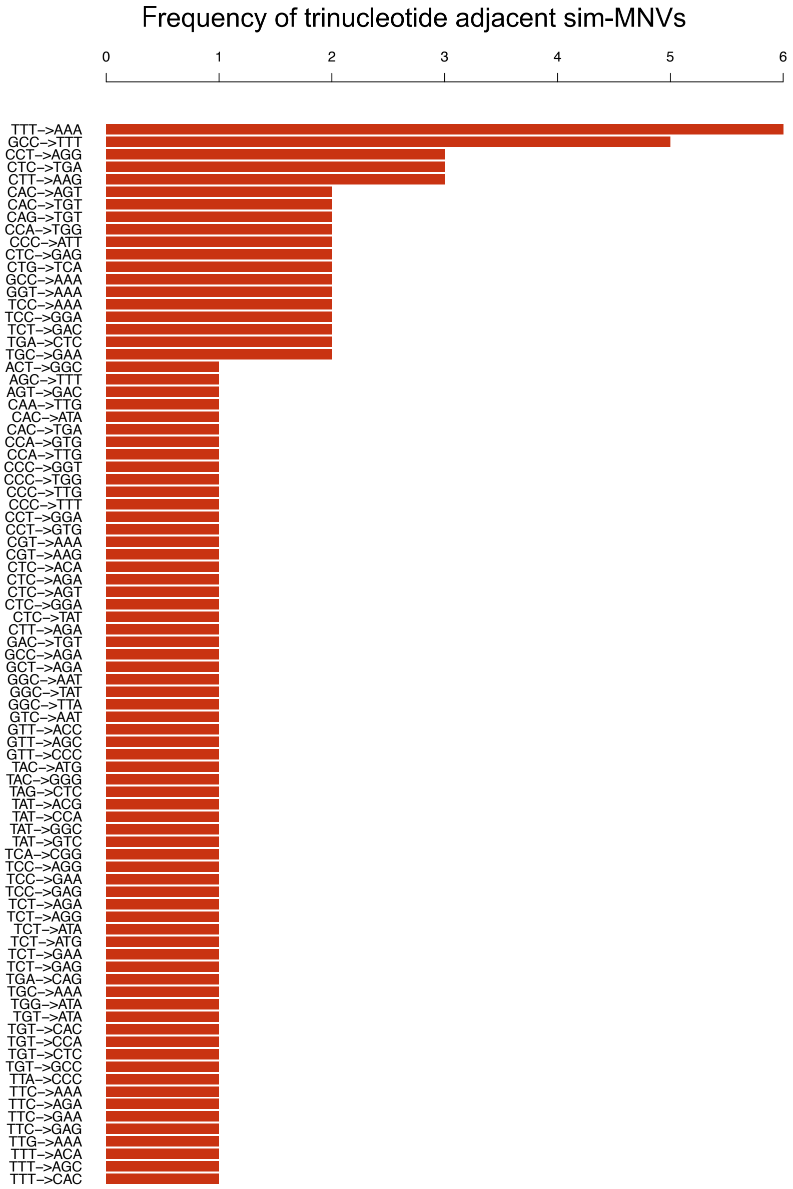


Figure S3: Mutational spectra of adjacent trinucleotide MNVs

Figure S4

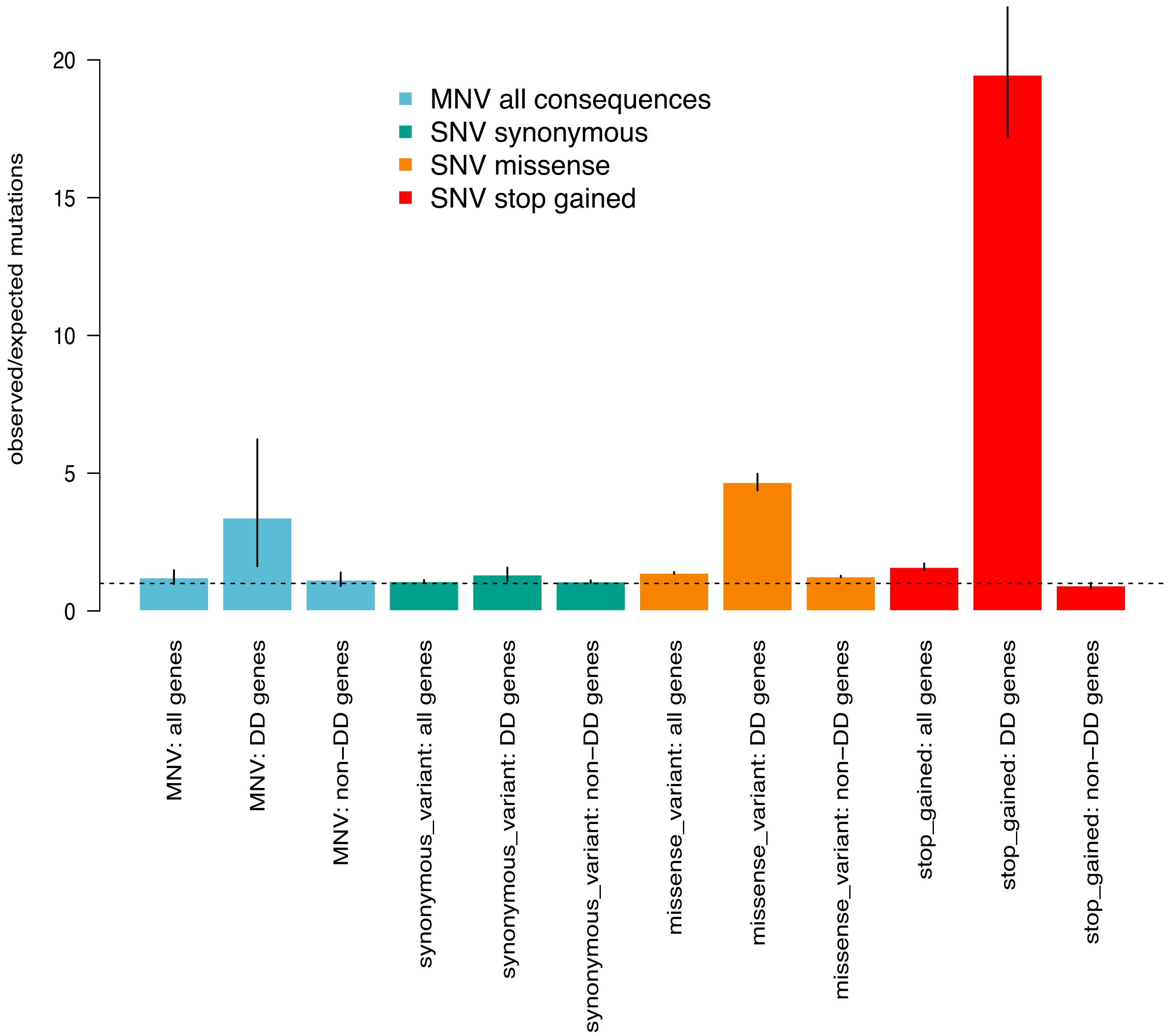


Figure S4 : Extended version of figure 5. Ratio of observed number of de novo MNVs vs the expected number based on the MNV mutation rate but comparing to a wider range of SNV enrichment including those not in DD genes by consequence.

Figure S5

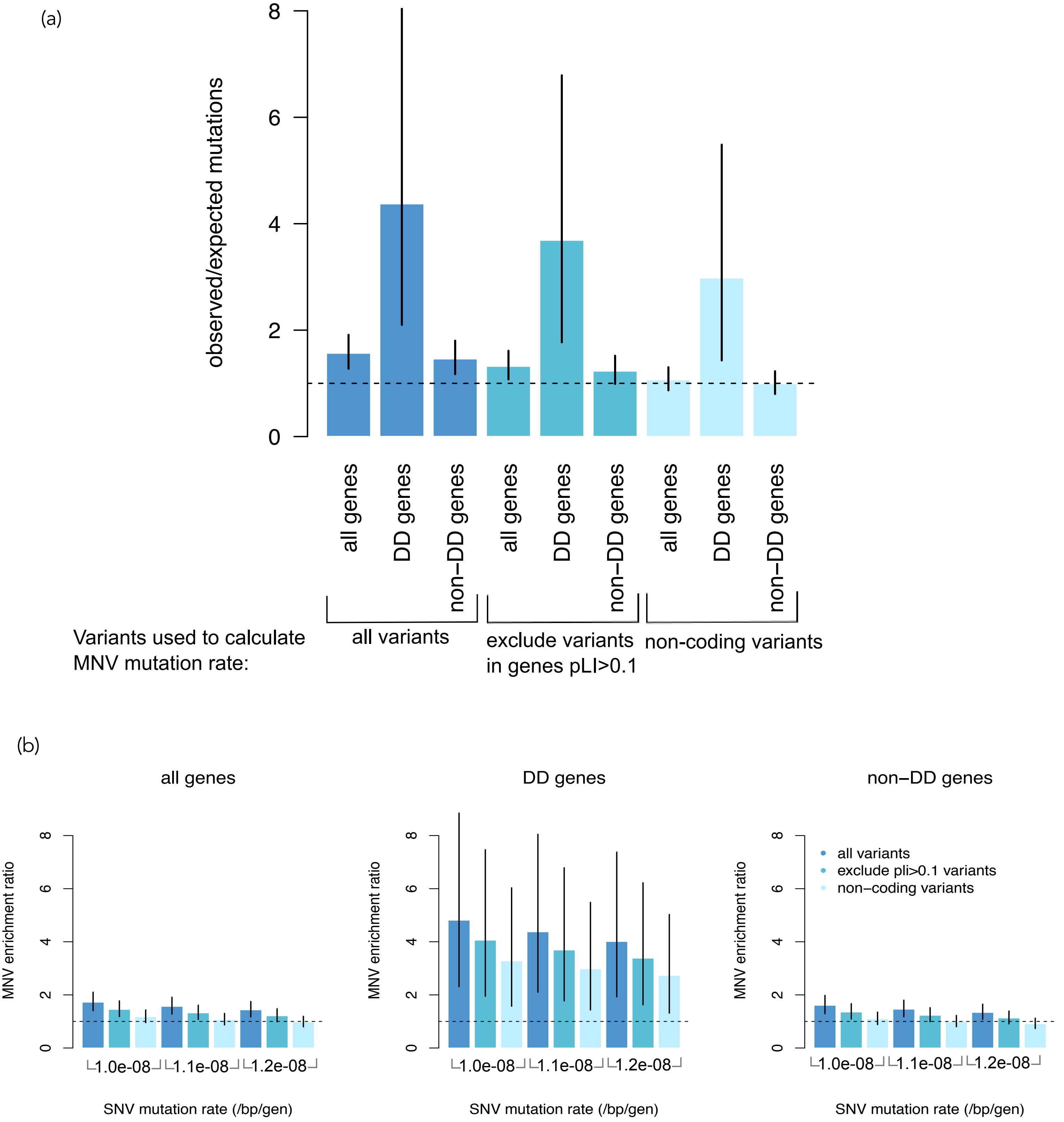


Figure S5: Sensitivity of MNV enrichment analysis to MNV mutation rate estimates (a) The impact of varying the subsets of variants used to estimate the MNV mutation rate estimate on the enrichment of de novo MNVs in different subcategories of genes as in Figure 5. These were all calculated using an SNV mutation rate estimate of 1.1x10-8 /bp/generation. (b) Using three different estimates of the SNV mutation rate estimate and the subcategories of variants as in (a) looking at the difference in enrichment ratios across the same subcategories of genes as in (a).

Table S1

| Analysis | Conclusion | Method | p-value |
|---|---|---|---|
| *Functional consequences of MNVs* | | | |
| Amino acid distance | Median amino acid distance is significantly larger for two-step than one-step missense MNVs | Wilcoxon Test | $1.1x10^{-7}$ |
| | Median amino acid distance for one step missense MNV is significantly larger than for exclusive SN missense changes | Wilcoxon Test | 0.0008 |
| Proportion of variants in highly constrained (pLI>0.9) genes | proportion of inter-codon $MNV_{1\text{-}20bp}$ that fall in highly constrained genes (pLI>0.9) is significantly smaller compared to missense SNVs | Proportion Test | 0.0007 |
| | proportion of two-step missense MNVs observed in highly constrained genes was also significantly smaller than for missense SNVs | Proportion Test | 0.0016 |
| | proportion of ExAC two-step MNVs in high pLI genes was significantly smaller than for ExAC missense SNVs | Proportion Test | $9.84x10^{-6}$ |
| CADD score | median CADD score for two-step missense MNVs was significantly higher than one-step missense MNVs | Wilcoxon Test | 0.00017 |
| | median CADD score for two-step missense MNVs was significantly higher than missense SNVs | Wilcoxon Test | $2.70x10^{-8}$ |
| Singleton Proportion | singleton proportion for two-step missense MNVs was nominally significantly higher compared to missense SNVs | Proportion Test | 0.02 |
| *Contribution of de novo MNVs to developmental disorders* | | | |
| De novo MNV enrichment | de novo MNVs were found to be significiantly enriched based on our estimated MNV mutation rate | Poisson Test | $1.03 \times 10^{-3}$ |
| | de novo MNVs were found to be significiantly enriched based on our estimated MNV mutation rate after correcting for sequence context | Poisson Test | $2.28 \times 10^{-3}$ |
| Undrepresentation in ClinVar | De novo MNVs were found to be depleted compared to expected in ClinVar | Poisson Test | $2.8x10^{-5}$, |

Supplemental Table 1: Summary of statistical tests performed in the analyses
